# Analysis of Cross-Cultural Communication in English Subjects and the Realization of Deep Learning Teaching

**DOI:** 10.1155/2022/4620738

**Published:** 2022-08-24

**Authors:** Jianing Niu, Yang Liu

**Affiliations:** ^1^School of Foreign Languages, Dalian Jiaotong University, Dalian, Liaoning 116028, China; ^2^Maritime History and Culture Research Center, Dalian Maritime University, Dalian, Liaoning 116026, China

## Abstract

In subject teaching, subject characteristics are the logical starting point for teaching development, and a deep understanding of subject characteristics is the basis for effective teaching. In practice, due to ignoring the cross-cultural understanding of middle school English subjects, there has been a long-term dilemma of “emphasizing knowledge and neglecting culture,” and overemphasis on the instrumental nature of middle school English has caused middle school English teaching to lose cultural self-cultivation and obscured the due education human value. Under the background of globalization and diversification, it is more and more important to develop students' cross-cultural awareness. How to realize cross-culturalism in middle school English teaching has become an important subject of teaching reform and development. From the perspective of English subject, this research constructs a scientific and implementable English listening and speaking teaching mode that promotes students' in-depth learning, provides specific and operational teaching implementation plans for English subject teachers in front-line teaching, and improves classroom teaching. It can promote students' construction of the meaning of language knowledge, stimulate their learning motivation, and finally achieve the common development of teachers and students.

## 1. Introduction

With the advancement of globalization in the world, major changes have taken place in the world economy, politics, culture, education, and other fields. The content of English education in our country has gradually changed from teaching English and American native languages to teaching international languages. Therefore, people understanding of English education needs to be updated. The era of globalization requires not only to change the way of thinking that we are accustomed to, but more importantly, to use it as an explanatory means and background for explaining social transformation and sociological theoretical changes. Only by understanding globalization can we have a deeper understanding of the development of English education in middle schools in our country under the context of globalization. The cultivation of cross-cultural competence in middle school English teaching advocates an equal and win-win cultural attitude, enhances students' ability to empathize, tolerate, and negotiate, and further improves students' ability to express local culture accurately and fluently. Some researchers describe this ability as “social cultural ability,” that is, the ability to use existing knowledge and skills to effectively process social and cultural information, so that the personality can develop in the direction of more integration and fuller potential. From English-based doctrine to cultural diversity, the cultivation of students' cross-cultural competence in middle school English education in our country is needed to update the understanding of middle school English education under the changing times. That is to say, it is necessary to carry out an in-depth contemporary understanding of English education in middle schools in our country: first, English education in middle schools in our country should be reflected in the learning of students' English language structure and the cultivation of language ability, and secondly, it can also promote middle school students through language learning, form a simple understanding of multicultural awareness, and help language learners to have a certain sense of belonging to the international society, as well as to have their own understanding and judgment of today's complex cultural integration. It can be considered that the purpose of cultivating students' cross-cultural competence in English education in secondary schools in our country is to promote students' respect and understanding of the cultural diversity of today's world, to appreciate the world's excellent cultures, and to deepen their recognition of their mother tongue culture [1–7]. This research constructs a teaching model of cross-cultural listening, speaking, listening and speaking in primary schools that can be implemented in the context of information technology. It consists of six parts: teaching preparation, setting up situations, activating prophets, acquiring new knowledge, in-depth knowledge processing, teaching evaluation and reflection. Finally, three rounds of action research were carried out on the model in the experimental class. After practice, the results were analyzed from four aspects: comparison of students' deep learning characteristics, students' learning effect, classroom observation, and teacher-student interviews to test whether the model has effectiveness.

## 2. Related Work

In recent years, research on cultural diversity, research on cross-cultural education, and research on cross-cultural teaching in English subjects have received more attention. Many studies focus on analyzing how foreign English teaching methods or models are combined with Chinese middle school English classroom teaching practice. It is certainly worthy of affirmation to continuously learn foreign advanced teaching concepts, but we must also see that the language application environment of foreign language teaching at home and abroad is different, the objective teaching conditions are different, and the difficulties and problems in the teaching process are also different. Researchers can seriously consider combining the actual situation and needs of English teaching in middle schools in our country and explore and think about the research road of English teaching in middle schools with Chinese characteristics. Secondly, it is necessary to pay attention to the relevant literature on cross-cultural teaching of English subjects. Cross-cultural teaching can be understood as the teaching of cultural factors and their contents in a cross-cultural environment, mainly in foreign language education. For example, the direct teaching method at the end of the nineteenth century began to incorporate cultural factors into the thinking scope of foreign language teaching. Linguists and cultural anthropologists began to pay attention to the cultural factors in foreign language teaching, and the inseparable connection between language and culture has become a more generally accepted view. In the 1950s, the symposium on the relationship between language and culture held by the American Foreign Language Education Association marked the birth of intercultural communication. The researchers at the conference reached a consensus that the cultural knowledge of the language country should be taught in the process of foreign language teaching. There is no doubt that this conference is a landmark conference in the field of foreign language teaching. Then in 1959, the anthropologist Hall first proposed the concept of “intercultural communication” in “Silent Language,” discussing the important role of culture in people's social life. Hall believes “Culture is the environment in which human beings live, and all aspects of human life are influenced by culture and change with cultural changes.” Cultural diversity education, as the development of multicultural education, involves cultural diversity, sexual policies, the main mode of education, education fairness, the inevitability of cultural diversity in the era of globalization, etc., among which the research on cultural diversity of ethnic cultural differences is relatively rich. The research of intercultural education mainly focuses on the concept, development background, goal, meaning, connotation, and practice of intercultural education in different countries. Intercultural education research provides a problem domain for intercultural teaching research. The research on cross-cultural teaching in English subjects mainly focuses on how to carry out cross-cultural teaching in English subjects. Among them, there are many studies from the perspective of linguistic second language acquisition, research on cross-cultural teaching methods, and cross-cultural learning in foreign language subjects. Research has received a lot of attention. Cross-cultural learning is also a research hotspot in recent years. Teaching is for learning. Therefore, when discussing the cross-cultural nature of English in middle school, it is inevitable to involve cross-cultural learning. Zheng Tainian discussed the various connotations and denotations of cross-cultural learning. The research believes that “cross-cultural learning occurs when a person learns from one cultural environment into another cultural environment” [8–13]. In a word, combined with previous researches on cross-cultural teaching of English at home and abroad, it can be found that there are the following characteristics: First, the research content is mostly concentrated on the investigation of the current situation of cross-cultural teaching, the cultivation of cross-cultural competence, and cross-cultural teaching, awareness training, and how to infiltrate cross-cultural teaching in middle school English teaching. Second, the research is relatively scattered, there is a certain degree of repetition, and there are few systematic studies. Third, foreign research on cross-cultural teaching is relatively mature, but how to combine it with the actual situation of Chinese middle school English teaching still requires researchers to continue to think.

## 3. Theoretical Basis of the Deep Learning Teaching Mode of English Cross-Cultural Listening and Speaking

### 3.1. Deep Learning Route

**Figure 1:**
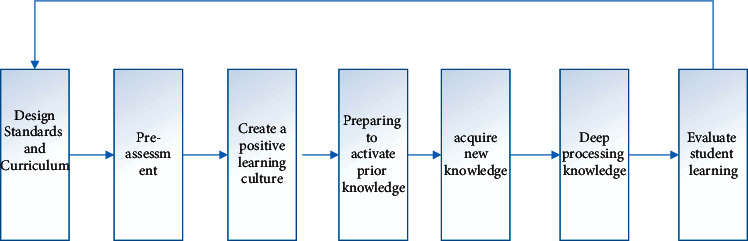
DELC deep learning route.

For the deep learning route American scholars Eric Jensen and LeAnn Nickelsen in their book “7 Powerful Strategies for Deep Learning” proposed a teaching model for teachers' reference in classroom teaching, referred to as DELC. The learning route is mainly composed of seven steps of designing standards and classrooms, preassessment, creating a positive learning culture, preparing to activate prior knowledge, acquiring new knowledge, in-depth processing of knowledge, and evaluating students' learning, as shown in the figure below. Among them, design standards and curriculum and preassessment are important preparatory stages for deep learning teaching. Teachers should understand the relationship between curriculum design and national curriculum and standards and clarify teaching content and goals. At the same time, preassessment is an important way to understand students' ability level. Teachers can carry out preassessment of teaching units or preassessment of individual goals. Creating a positive learning culture has a positive guiding effect on students' positive emotional promotion and learning engagement in the classroom. In the process of deep learning, knowledge is not fragmented. Teachers should use appropriate teaching methods such as questioning and discussion to promote students to activate prophets and form connections, so as to better obtain the input of new knowledge and realize the connection and integration of old and new knowledge. Deep processing knowledge is the core part of the deep learning route. It is a field that promotes learners to achieve fine and effective processing. It can be achieved through four links: awareness, analysis and synthesis, application, and assimilation. The final evaluation of students' learning is the summary feedback of the entire learning process. This step can be implemented in the form of self-reflection, peer feedback, etc., so as to further understand the students' ideas and provide important references for subsequent teaching, as shown in Figure 1 [14].

### 3.2. Deep Learning Cognition

**Figure 2:**
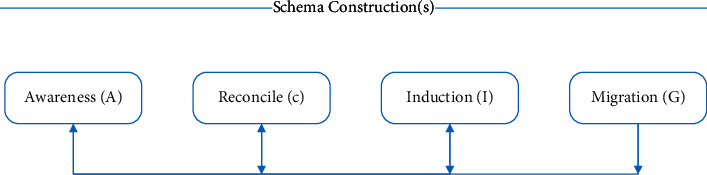
S-ACIG deep learning cognitive process.

S-ACIG cognitive process is a deep learning cognitive process proposed by Professor Hu Hang through theoretical analysis of ACT-R, APOS, etc. and empirical operation research, which provides important theoretical guidance for the exploration of deep learning teaching practice. From the perspective of the learner, the cognitive process of S-ACIG deep learning involves four stages of awareness, reconciliation, induction, and transfer, and the schema runs through the whole process of learning, as shown in Figure 2 below. S-schema means that the cognitive process of deep learning is the process of schema construction. A-Awareness is the initial stage of learning. Learners perceive through different teaching activities or situational presentations, import declarative knowledge into the memory system, and understand the phenomenon they feel. C-Reconciliation is a method by which learners can put forward a preliminary solution to a problem through analysis and thinking when they encounter a problem for the first time, which is a process involving coordination between two or more programs. I-Induction is a process in which learners absorb and edit knowledge on the basis of reconciliation, transform declarative knowledge into procedural knowledge, and form a reasonable cognitive structure. G-transfer is that when learners master relevant knowledge, they form a stable cognitive structure system and can transfer to different situations and problem solving. At the same time, in this process, the existing schemas are constantly revised and improved, and variant exercises are carried out through comprehensive use, thereby improving their problem-solving ability.

### 3.3. Common Teaching Modes of English Cross-Cultural Listening and Speaking

**Figure 3:**
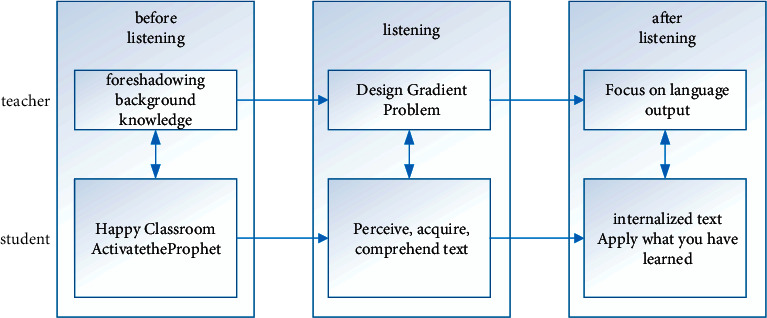
English cross-cultural listening and speaking teaching mode.

In the traditional English teaching mode, teachers will pay more attention to imparting knowledge to students, ignoring the subject status of students, so that students become passive recipients of knowledge. With the continuous updating of teaching concepts, educational researchers are also exploring new teaching modes. For example, the application of PWP teaching mode in cross-cultural English listening, speaking, reading, and writing teaching is more common today. In English cross-cultural listening and speaking teaching, PWP is mainly reflected in three teaching stages: prelistening (before listening), while-listening (in listening), and postlistening (after listening). Design teaching tasks and activities to promote the improvement of students' language knowledge and ability. For example, we have explored the use of PWP teaching mode to carry out cross-cultural English listening and speaking teaching and divided teaching into three stages: before listening, during listening, and after listening. The model is shown in Figure 3 below [15]. Before listening, teachers lay the foundation for background knowledge and activate students' existing knowledge; design gradient problems during listening to promote students' perception of text; after listening, focus on language output and guide students to apply what they have learned. It also pointed out that teachers should base themselves on the text and make in-depth analysis in the teaching of English cross-cultural listening and speaking; design the overall goal and clarify the main line; promote students to be good at listening and cultivate students' good study habits. This provides an important reference for the optimization of the subsequent English cross-cultural listening and speaking teaching mode.

### 3.4. Teaching Mode of Deep Learning of English Cross-Cultural Listening and Speaking

**Figure 4:**
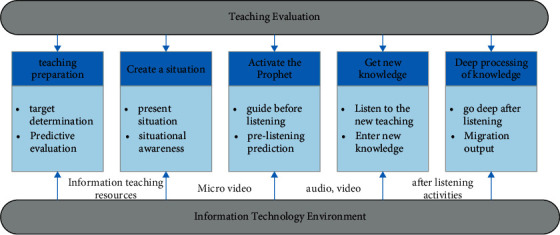
Teaching mode of cross-cultural listening, speaking, and deep learning in primary school English.

This study is based on deep learning related theories and research, combined with foreign deep learning routes, S-ACIG deep learning cognitive process, and common teaching modes of English cross-cultural listening and speaking. The training objectives of the primary school English cross-cultural listening and speaking course and the actual situation of English classroom teaching have constructed a cross-cultural listening and speaking teaching model that promotes the in-depth learning of primary English in the information technology environment, as shown in the figure below. This model is mainly composed of six parts: teaching preparation, creating situation, activating prophets, acquiring new knowledge, deep processing of knowledge, and teaching evaluation and reflection, specifically as shown in Figure 4 [16].

## 4. Construction of a Cross-Cultural Listening and Speaking Teaching Model for English Deep Learning

### 4.1. Teaching Preparation

#### 4.1.1. Determination of Teaching Objectives

English Curriculum Standard also points out that the English courses in the entire basic education stage are divided into nine levels of target systems according to their ability levels. The compulsory education stage needs to reach the fifth level of ability, while the sixth grade of primary school students needs to reach the second level of ability. At the same time, the objectives at all levels of the course are set from five aspects: language skills, language knowledge, emotional attitudes, learning strategies, and cultural awareness, but the teaching objectives in classroom teaching can generally be divided into three aspects: knowledge, skills, and emotional attitudes to be set according to the teaching content. When setting teaching goals, teachers must connect with reality, refer to the ability level requirements of each stage, and conform to the students' proximal development zone, so as to better stimulate students' enthusiasm and guide students into a real learning state. In addition, in the teaching of cross-cultural listening and speaking courses, teachers should set oral language output and expression ability requirements that meet the students' conditions according to the corresponding content of the textbooks, so as to achieve the ability level of promoting speaking and cross-cultural listening and speaking, rather than blindly completing the listening section, so as to further promote the occurrence of deep learning in English cross-cultural listening and speaking classrooms [17]. Therefore, in the process of deep learning teaching, teachers should set reasonable and appropriate teaching goals based on the actual situation of students, teaching content, curriculum requirements, and characteristics of deep learning, so as to make themselves more clear about how to learn, while enabling students to establish clear learning goals and learning awareness.

#### 4.1.2. Predictive Evaluation

Predictive evaluation is an important way to understand students' mastery of learning and to adjust classroom teaching goals in a timely manner. It can evaluate and test a certain teaching unit and can also evaluate and test a certain section of teaching content. It should be noted that the prediction evaluation does not need to be very difficult. It is mainly to detect the mastery of students' knowledge and to warm up for the subsequent teaching work, with positivity. For teachers, in order to further promote students' in-depth learning in English cross-cultural listening and speaking classes, teachers can simply and quickly understand the students' previous knowledge and the existing reserves of the content to be taught by issuing a small bill of lading; or you can also borrow PPT courseware to display core questions and interact with students to review, preassess, and so on through question-and-answer interactions. Through a short preclass prediction evaluation, teachers can not only understand the students' mastery of the content of the previous class, but also conduct pretesting of the knowledge points to be taught in this class to understand the students' current knowledge background and recent development. The situation of the district can further give feedback about whether the existing teaching goals match the current situation of the students. If the difference is obvious, the follow-up teaching can be effectively adjusted in time to ensure the teaching quality and efficiency of the classroom. At the same time, through the teacher's problem detection and guidance, students can better consolidate the knowledge they have learned and arouse their own experience related to the knowledge to be taught in a relaxed atmosphere, prepare for the following learning content, and stimulate inner learning positivity [18].

### 4.2. Create a Learning Situation

**Figure 5:**
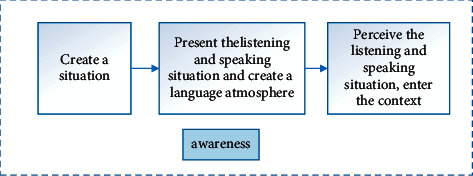
Flowchart of creating a scenario.

As shown in Figure 5, creating an appropriate learning situation is an indispensable and important way in English teaching. Language learning itself is closely related to various life situations. Interesting and practical learning situations can create a good language learning atmosphere and stimulate students to learn. The initiative and enthusiasm of deep learning also provide more feasibility for the occurrence of deep learning. In the information technology environment, teachers have greater convenience and wider choice for the creation of learning situations. It is not difficult to find that the current primary school students' attitude towards English cross-cultural listening and speaking courses is still relatively positive, which creates favorable conditions for the occurrence of in-depth English cross-cultural listening and speaking courses [19]. Therefore, when creating a situation, teachers should use information technology to start more from the perspective of students, fit students' life experience and cognitive ability, and design real life situations in combination with teaching content that is more attractive and resonates with students. For example, when learning ways to go to school, teachers can show pictures or related videos of students' daily transportation to and from school. When learning our favorite season, teachers can show students' familiar campus four seasons on the multimedia courseware, and the will students feeling related to it will increase your inner motivation to participate. At the same time, we all know that interest is the best teacher for students to learn, so if the teacher creates an appropriate situation, the interest of the students will be stimulated, and they can connect with similar experiences in their own life in combination with the situation provided by the teacher. Under the guidance of teachers, think and distribute thinking. In this process, the students are not passive receivers, but more on exerting their own subjective initiative, perceiving the situation, connecting the situation with their own experience, generating curiosity, and following the teacher's guidance to enter the follow-up step-by-step study.

### 4.3. Activate Old Knowledge

**Figure 6:**
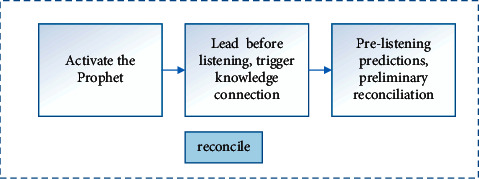
Activating prophet flowchart.

As shown in Figure 6, the old knowledge is the prophet, that is, the knowledge that students have in their minds, including the knowledge gained from previous teaching and their own knowledge and experience. Activating old knowledge is an important part of promoting deep learning. In daily teaching, teachers can easily ignore the activation of old knowledge and directly teach new knowledge. Teachers can effectively guide students to activate the prophet, which can prompt students to recall and consolidate the knowledge they have learned, connect and integrate the previous knowledge with the new knowledge to be learned, so as to promote the understanding and mastery of the new knowledge. Deep learning emphasizes the connection and integration between old and new knowledge. Therefore, in the process of in-depth English cross-cultural listening and speaking, teachers should combine the previous teaching content with the teaching content of this class and classroom teaching goals to determine how to activate students' prophets, activate students' prior knowledge in which areas, and so on. Here, teachers can synthesize the actual situation of the students during the previous preassessment. For example, in the teaching of the cross-cultural listening and speaking class, teachers can use the teaching content of the vocabulary class in the previous section and the connection between the knowledge in the vocabulary class and the content of the cross-cultural listening and speaking class and interpret words, phrases, and sentences with related parts. The expression is activated through question guidance, and at the same time, with the assistance of the situation, the teacher guides before listening, so that students will not feel at a loss when they complete listening tasks and learn new knowledge but establish a process of transition. On this basis, the teaching of new knowledge will give students a certain amount of preparation in knowledge and psychology, and under the guidance of teachers, students can predict the listening content before listening and have a certain perception of core vocabulary. This makes it easier to complete listening teaching and tasks. In addition, when students successfully extract the prior knowledge, the input of subsequent new knowledge can also establish a better connection, thereby promoting their own understanding of the new content [20].

### 4.4. Acquiring New Knowledge

**Figure 7:**
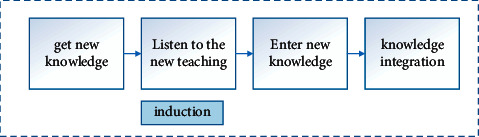
Flowchart of acquiring new knowledge.

As shown in Figure 7, acquiring new knowledge is a key part for students to input new knowledge content, and it is also the basic condition for deep learning to occur. Teaching new knowledge will be easier when teachers create relevant situations and guide students to activate prophets. The acquisition stage of new knowledge in deep learning is not only about learning new content, but also on the basis of learning new knowledge, and then it establishes a connection between new and old knowledge and further inducts and integrates it, so as to internalize and form its own cognitive structure. When teachers teach new knowledge, they should abandon the traditional indoctrination teaching mode, change the state of students passively accepting knowledge, implement classroom teaching for all students, reflect the idea of teacher-led, student-centered, and give full play to students' learning ability, with autonomy. In English cross-cultural listening and speaking teaching, teachers should focus on the teaching process, rather than blindly getting answers to questions. It can be seen from the previous survey on the current situation of English in-depth learning that the connection and integration ability of students' knowledge is still relatively weak, and most students are still unable to integrate and output new and old knowledge. Therefore, in the teaching of cross-cultural listening and speaking, teachers should design the teaching process according to the real situation of students, arrange teaching activities in line with students' age and physical and mental characteristics, and guide students to fully participate in the classroom. And in listening teaching, teachers should design listening tasks progressively layer by layer, from easy to difficult, combined with front and back, such as listen and tick, listen and fill the blank, listen and choose, retell the text, and other tasks, step by step to promote students' understanding of listening materials. In this process, the teacher can give the students appropriate prompts when necessary, so as to guide the students to say the correct answer, so that the students will gradually understand the content of the listening materials and input new knowledge and establish relevant connections for induction and integration.

### 4.5. Deep Processing of Knowledge

**Figure 8:**
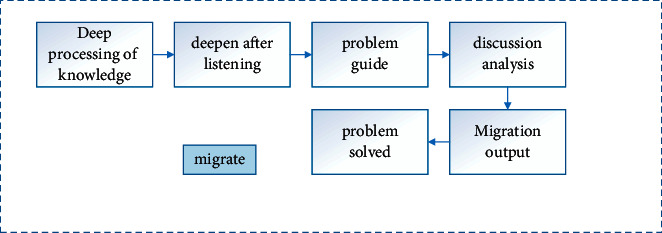
Flowchart of knowledge deep processing.

As shown in Figure 8, the deep processing of knowledge is the core part of deep learning, and it is also a key step to promote deep learning for students. The in-depth processing of knowledge is the stage of deepening after listening after students have initially input new knowledge. In this stage, teachers can design postlistening activities to promote students' consolidation and deepening according to different cross-cultural listening and speaking teaching contents, so as to promote the improvement of students' language output, peer cooperation, transfer application, problem solving, and other abilities. In the in-depth processing stage of English cross-cultural listening and speaking teaching, teachers can design output activities in different forms such as pair work, team work, role play, and having a speech/presentation. What we should pay attention to here is that language learning is not the training of a single language skill, but the comprehensive application of multiple language skills such as listening, speaking, reading, and writing. English learning focuses on outputting exercises after a large amount of input learning, so as to truly realize the communicative function of the language. Therefore, after completing the listening task, the output activities of comprehensive cross-cultural listening and speaking exercises should be further designed. For example, in addition to basic exercises such as listening and reading, role reading, etc., situation reconstruction, investigation reports, and speeches around topics can be set up along with other creative activities. Of course, listening, speaking, reading, and writing can also be combined to further promote the improvement of students' language ability. In the previous survey, we can see that the output ability of students in cross-cultural listening and speaking teaching is very weak, which is also largely related to the fact that teachers ignore this part in daily teaching [21]. Therefore, in the process of in-depth learning, teachers should at least combine the two abilities of cross-cultural listening and speaking, create a real and interesting situation, design corresponding practice tasks, and promote students to have a deeper discussion and analysis through questions. For the transfer output of listening teaching content, the teacher evaluates and summarizes the completion of the students and gives timely feedback to the students, so that deep learning can really take place. At the same time, under the guidance of teachers, students will further consolidate and deepen the newly input knowledge, transfer and apply the knowledge they have learned, and integrate the output, so as to continuously improve their problem-solving ability and language application ability.

### 4.6. Teaching Evaluation

Assessment is an important part of every course, and English subjects are no exception. The evaluation of English courses should be based on the corresponding course teaching objectives, using reasonable and scientific evaluation methods and diversified evaluation methods, to monitor the teaching process and teaching results in a timely and effective manner, so as to play a positive guiding role in teaching. In the process of English cross-cultural listening and speaking in-depth learning, this research adopts the methods of teacher evaluation, peer evaluation, and student self-evaluation, combined with formative evaluation and summative evaluation to conduct comprehensive and scientific monitoring of teaching [22].

### 4.7. Information Technology Environment

The information technology environment, as the background support of the whole teaching mode of English cross-cultural listening and speaking deep learning, also plays an important role in promoting the occurrence of students' deep learning. In the teaching preparation stage, teachers can use the WeChat platform to send teaching resources such as animation videos related to the teaching content to students, so that students can watch and learn independently in advance and warm up the knowledge points to be learned, so that they can enter the classroom more quickly with learning state, leaving more time for the in-depth processing of teaching. In the stage of creating a situation, teachers should actively use modern educational technology, combined with teaching content, create a logical cross-cultural English listening and speaking teaching situation through video, pictures, animation, audio, etc., and turn abstraction into an image, so as to guide students to actively participate. In the classroom teaching, the initiative and investment of students' follow-up learning can be further improved. In the two stages of activating old knowledge and acquiring new knowledge, teachers can use multimedia courseware to help students activate prophets through question chains or picture guidance with the subject. In addition, in the deep processing stage of knowledge, the support of information technology also plays a key role in the occurrence of students' deep learning. Teachers can use multimedia computers to design output activities and provide students with a display platform for output applications after listening, such as the use of interesting dubbing, so as to further promote students' in-depth processing and practical application of cross-cultural listening and speaking skills. All in all, under the support of the information technology environment, English cross-cultural listening and speaking teaching can effectively break the constraints of traditional teaching, making teaching more abundant, flexible, authentic, and practical, and at the same time it is easier to promote the occurrence of students' deep learning.

## 5. Practice and Result Analysis of Deep Learning Teaching Mode of English Cross-Cultural Listening and Speaking

### 5.1. Preliminary Preparation for Teaching Practice

#### 5.1.1. Teaching Preparation

The teaching subjects selected in this study are still relatively enthusiastic about their attitude towards learning English and have a strong desire to learn after a preliminary investigation. Students at this stage are highly curious and active. They have mastered some common English language knowledge and have certain listening and speaking skills. But generally speaking, students' listening and speaking skills still need to be learned.

#### 5.1.2. Teaching Content

The source of the teaching materials for the practice of listening and speaking teaching mode for in-depth learning of primary school English in this study is the “Compulsory Education Textbook English (Starting Point for Grade 3)” coedited by the People's Education Press, the English Curriculum Textbook Research and Development Center of the Curriculum Textbook Research Institute, and Canada Lingtong Education Co., Ltd. “Grade 6—Book 1.” This book consists of six teaching units and two review units in total. Each teaching unit is divided into three parts, A, B, and C. Parts A and B are the main teaching part, and part C is the flexible teaching content for teachers to choose.

### 5.2. The Specific Implementation of Teaching Practice

#### 5.2.1. Planning Phase

**Figure 9:**
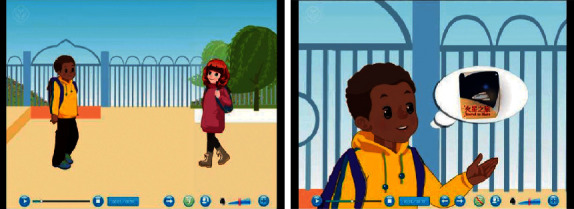
Example of preclass teaching resources.

Teaching preparation is mainly based on the teaching content and the actual situation of students to determine the teaching objectives of this course and the corresponding teaching courseware and other preparations. This round of presentation is based on the let's try and let's talk listening and speaking teaching section of part B of “My weekend plan” 3 units as an example. Before teaching, teachers can send teaching resources such as animation videos related to this teaching section to the WeChat class group, so that students can watch and study them one day in advance. It can effectively shorten the transition time when learning in the main class, so as to enter the learning state faster, as shown in Figure 9.

  Knowledge goals: Be able to understand and master the gist of the dialogue; read the text according to the correct pronunciation and intonation; be able to correctly distinguish the usage and meaning of where and when, and be able to use sentence patterns in situations where are/is …going? we're/I am going to …; when are/is …going? We're/I'm going … talking about time and place. Skill goal: be able to understand the requirements of the questions and predict the key content of the listening materials before listening; be able to infer the subsequent story development after completing simple exercises; be able to use basic listening skills to complete the corresponding themes and details under the guidance of the teacher; questions: can make oral output expressions on related topics based on listening key content.
  Emotional goals: learn common sense about movie theaters and ticket prices through dialogue; experience teamwork spirit through cooperation and interaction in the learning process.

**Figure 10:**
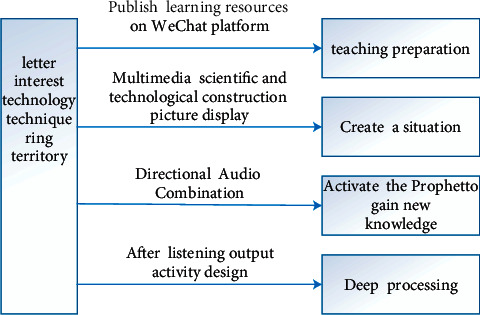
Information technology support function diagram.

In this study, the information technology environment, as the key background support for the entire deep learning teaching model, also plays an important role in the teaching practice process. In the first round of action research, the supporting role of information technology in each link of this round of teaching practice is mainly reflected in the following aspects as shown in Figure 10.

#### 5.2.2. Action and Observation Phase

In this session, the teacher created a corresponding situation based on the teaching content. First, he used the multimedia courseware to show the pictures of the main characters in the following text and then asked who to ask questions. Then the teacher presented the scene where John met Amy and chatted on the way home. The scene guides students with questions, allowing students to guess the content of the scene, so as to prepare for formal dialogue learning. Teachers can effectively stimulate students' curiosity, enhance students' interest in learning by presenting real situations and guide them with questions, and create a language environment and atmosphere for follow-up dialogue learning.

Make good use of the situation created earlier, and further disperse students' thinking by asking questions, promote their extraction of existing knowledge in their minds, stimulate students' initiative in learning, and allow students to think and explore. Here, you can also preliminarily reconcile students' knowledge through simple questions in the let's try part. While reviewing the content of the let's learn part of this unit through question and answer, you can also prepare for the listening and speaking part of the let's talk later. The characters and scenes are the foreshadowing, and students are allowed to infer the development of the subsequent plot based on the existing information, so that there is a transition between psychology and knowledge, and it will be easier to understand and receive the following content.

When teachers activate the students' prophets through the warm-up session of let's try and let students reconcile the old and new knowledge, they can further start the formal teaching of the let's talk part. In this section, teachers set different levels of questions through computer courseware, throughout the entire listening practice, so that students can gradually understand the listening content in the process of completing the task and finally lead the students to explain the important knowledge points in the listening text, so that the students can summarize the relevant knowledge. Sort out, form your own internal knowledge, and prepare for the in-depth processing of subsequent knowledge.

**Figure 11:**
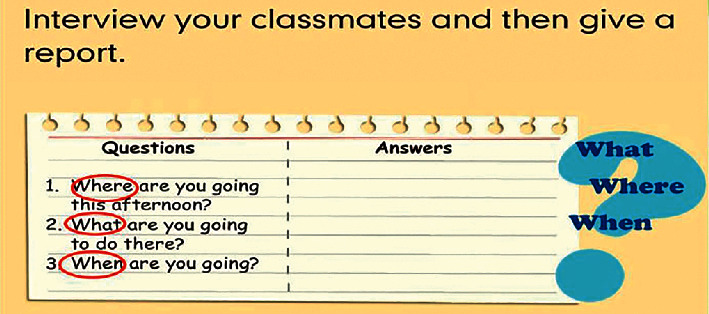
Example of activity output teaching.

In daily teaching, many teachers end the class after teaching knowledge and do not pay attention to guiding students to further transfer and apply knowledge, so that students' mastery of knowledge often stays on the surface and will be forgotten soon. The in-depth processing of knowledge is the key to promoting students' in-depth learning. After leading students to input new knowledge, teachers should guide students to further consolidate and deepen and convert the previous knowledge input into subsequent application output, that is, pay attention to students. In this link, teachers can design some interesting and cooperative output tasks. While transferring and applying knowledge, they can also exercise students' oral expression ability and truly achieve the combination of listening and speaking and listening, as shown in Figure 11.

#### 5.2.3. Evaluation Stage

This stage is a multilevel evaluation. First, teacher evaluation has been reflected in the previous teaching process. Teachers give timely feedback evaluation during the activity process and at the end of the activity. Most of them are based on motivational evaluation. Help students build confidence in learning and have the courage to express themselves. Secondly, in the first round of practice, students' self-evaluation and peer evaluation adopted a part of students who were selected to speak after the lecture. Most of the students' evaluation content was good, but some students reported that the learning process was not serious enough, and some knowledge points were not mastered in place.

## 6. Conclusion

With the rapid development of information technology and the abundance and change of knowledge, traditional learning methods can no longer effectively absorb and integrate contemporary information. We are now in the era of education informatization, which has new characteristics such as fast dissemination, huge amount of information, and knowledge explosion challenge. This requires us to break the traditional cramming-style teaching and single emphasis on knowledge transfer and should shift from the basic knowledge reserve to the stage of comprehensive ability development, so as to promote the development of learners' higher-order thinking, the meaning construction of knowledge, and the transfer and application of knowledge, and so on, so as to realize the deep learning and understanding of knowledge. Therefore, in recent years, researches related to deep learning have received much attention and have gradually become a hot topic in the field of subject teaching. Based on deep learning theory, second language acquisition theory, and constructivism theory, this study takes sixth-grade students as experimental objects to study the current situation of deep learning in English listening and speaking class and how to promote students to effectively develop deep learning. The author firstly conducted a survey on the current situation of deep learning in English listening and speaking course of students in the form of a questionnaire; on this basis, in order to further explore how to promote the effective occurrence of students' deep learning, this study also constructed a teaching model of primary school English listening and speaking deep learning. Apply it in practice and test the effectiveness of the model through practical teaching.

## Data Availability

The dataset can be accessed from the corresponding author upon request.
